# In Vivo Toxicity, Redox-Modulating Capacity and Intestinal Permeability of Novel Aroylhydrazone Derivatives as Anti-Tuberculosis Agents

**DOI:** 10.3390/pharmaceutics15010079

**Published:** 2022-12-26

**Authors:** Violeta Valcheva, Rumyana Simeonova, Milka Mileva, Stanislav Philipov, Reneta Petrova, Simeon Dimitrov, Almira Georgieva, Elina Tsvetanova, Yoana Teneva, Violina T. Angelova

**Affiliations:** 1 The Stephan Angeloff Institute of Microbiology, Bulgarian Academy of Sciences, 1113 Sofia, Bulgaria; 2 Department of Chemistry, Faculty of Pharmacy, Medical University of Sofia, 1000 Sofia, Bulgaria; 3 Department of Human Anatomy, Histology, General and Clinical Pathology and Forensic Medicine, Faculty of Medicine, Sofia University “St. Kliment Ohridski”, 1407 Sofia, Bulgaria; 4 National Diagnostic and Research Veterinary Medical Institute, 1000 Sofia, Bulgaria; 5 Laboratory of Free Radical Processes, Institute of Neurobiology, Bulgarian Academy of Sciences, 1113 Sofia, Bulgaria

**Keywords:** aroylhydrazone derivatives, acute and sub-acute toxicity, redox-modulating capacity, GIT permeability, pathomorphological evaluation

## Abstract

The emergence and spread of *Mycobacterium tuberculosis* strains resistant to many or all anti-tuberculosis (TB) drugs require the development of new compounds both efficient and with minimal side effects. Structure-activity-toxicity relationships of such novel, structurally diverse compounds must be thoroughly elucidated before further development. Here, we present the aroylhydrazone compounds (**3a** and **3b**) regarding their: (i) acute and subacute toxicity in mice; (ii) redox-modulating in vivo and in vitro capacity; (iii) pathomorphology in the liver, kidney, and small intestine tissue specimens; and (iv) intestinal permeability. The acute toxicity test showed that the two investigated compounds exhibited low toxicity by oral and intraperitoneal administration. Changes in behavior, food amount, and water intake were not observed during 14 days of the oral administration at two doses of 1/10 and 1/20 of the LD_50_. The histological examination of the different tissue specimens did not show toxic changes. The in vitro antioxidant assays confirmed the ex vivo results. High gastrointestinal tract permeability at all tested pH values were demonstrated for both compounds. To conclude, both compounds **3a** and **3b** are highly permeable with low toxicity and can be considered for further evaluation and/or lead optimization.

## 1. Introduction

Tuberculosis (TB) is among the top ten death causes, the leading cause of death from a single infectious agent and a leading cause of death of HIV-positive patients [1]. The emergence of drug resistance is a major threat to global TB control. The World Health Organization (WHO) estimates that up to half a million new cases of multidrug-resistant tuberculosis (MDR-TB) cases (i.e., resistant to, at least, rifampicin and isoniazid) occur each year globally. Current treatment regimens for MDR-TB are far from satisfactory: the overall duration is 20 months or more, requiring daily administration of drugs that are more toxic and less effective than those used to treat drug-susceptible TB and have a high cost. Alas, the newly approved drugs for treating multidrug-resistant tuberculosis (e.g., bedaquiline, delamanid, and pretomanid) in combination with other anti-TB drugs produce serious side effects, including hepatotoxicity, CNS toxicity, and cardiac dysrhythmia. Isoniazid administration has also been associated with numerous adverse consequences, affecting especially patients’ blood and liver [2]. This situation justifies the search and development of new compounds with anti-TB activity. Although biological activity is a key issue in the drug development process, ADME (absorption, distribution, metabolism, and excretion) properties of compounds are crucial no less. Poor pharmacokinetics is one of the primary reasons for the failure of drug candidates [3,4]. Since ADME processes are highly dependent upon the ability of chemicals to cross biological membranes, examination of permeability through gastrointestinal (GIT), hepatocyte membrane, target cells, blood-brain (BBB) barrier, etc. is of top importance [5].

In this context, Parallel Artificial Membrane Permeability Assay (PAMPA) provides a simple and cost-effective approach for the estimation of drug permeability potential [6]. Data for transcellular passage of large compound sets are generated in short terms that identify the candidates with favorable ADME profiles and support the effectiveness of further development workflow [7]. Since the oral route of administration is preferred for most drugs due to convenience, patient compliance, and cost concerns [8], in the present study PAMPA is employed to evaluate the GIT permeability of the newly synthesized isoniazid derivatives.

Hydrazones and aroylhydrazones are organic compounds classified as Schiff bases, with important antimycobacterial, antitumoral, antiviral, and antiparasitic activities [9,10,11,12]. Compounds with hydrazone and N-acylhydrazone groups also have important anti-inflammatory properties [13], an important characteristic for the pathogenesis of TB. To improve the activity of some of these against specific pathogens, such as *M. tuberculosis*, several research groups have conjugated them with drugs already used for TB treatment. Great results were obtained with Isoniazid (INH) derivatives, giving rise to selective and promising compounds [14,15,16,17,18,19,20,21]. 

**Figure 1:**
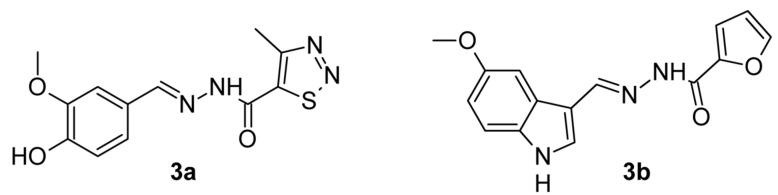
Structure of hydrazone derivatives **3a** and **3b**.

In our previous works [22,23] we performed: (i) design and synthesis of 28 novel isoniazid derivatives comprising hydrazone and sulfonyl hydrazone moiety. (ii) evaluation of their activity against *M. tuberculosis* H37Rv reference strain, and (iii) evaluation of their toxicological profile. Most of the tested compounds demonstrated low minimal inhibitory concentrations (0.07 to 2.9 µM), comparable to or lower than that of isoniazid, along with low cytotoxicity and a highly selective index (SI) [22,23]. All compounds were subjected to ADME/Tox computational predictions, which showed good bioavailability and fraction absorption and no risk of toxicity. Two of them, **3a** and **3b** (Figure 1), exhibited the highest antimycobacterial activity and minimal associated cytotoxicity against normal cell lines as follows: **3a**, (MIC = 0.0730 µM, cytotoxicity—HEK-293T IC_50_ = 256.7 µM, SI = 3516) [23] and **3b**, (MIC = 0.4412 µM, cytotoxicity—HEK-293T IC_50_ = 279.5 µM, SI = 633.49) [22]. The suitable scores of molecular docking performed on two crystallographic structures of enoyl-ACP reductase (InhA) provide promising insight into possible interaction with the InhA receptor [23].

In this sense, this study aimed to evaluate the toxicity risks, redox-modulating capacity, and intestinal permeability of the above aroylhydrazone compounds using comprehensive in vivo and in vitro methods.

## 2. Materials and Methods

### 2.1. Chemistry 

The chemicals and reagents employed in the synthesis of compounds were purchased from Sigma-Aldrich (Merck KGaA, Darmstadt, Germany). Hydrazones **3a** and **3b** were prepared by the condensation reaction of a 4-methyl-1,2,3-thiadiazole-5-carbohydrazide **1a** or furan-2-carbohydrazide **1b** and aldehydes vanillin **2a** or 5-methoxy-1H-indole-3-carbaldehyde **2b**, at a molar ratio of 1:1, in abs. ethanol for 1–2 h. The synthesized compounds: *N*′-[(4-hydroxy-3-methoxyphenyl)methylidene]-4-methyl-1,2,3-thiadiazole-5-carbohydrazide (**3a**) [23] and *N*′-[(5-methoxy-1*H*-indol-3-yl)methylidene]furan-2-carbohydrazide (**3b**) [22] were confirmed by ^1^H NMR, ^13^C NMR, and HRMS spectral data. The spectral analyses were carried out by the assigned structures.

### 2.2. Experimental Animals

Male and female Jcl:ICR mice (6 weeks old, 25–30 g) obtained from the National Breeding Center, Sofia, Bulgaria were used in the experiments. As the more sensitive sex [24], 36 females were used in the acute toxicity test and 36 males in the short-term toxicity test. Mice were housed in plexiglass cages (6 per cage) in a 12/12 light/ dark cycle under standard laboratory conditions (ambient temperature 20 ± 2 °C and humidity 72 ± 4%) with free access. to water and standard pelleted food No. 53-3, produced following ISO 9001:2008. Before the start of the experiment, mice were acclimatized to vivarium conditions for seven days and their health was monitored daily by a veterinarian. All experiments strictly adhered to the principles set out in the European Convention for the Protection of Vertebrate Animals Used for Experimental and Other Scientific Purposes (ETS 123) [25]. Efforts have been made to minimize animal suffering. The study was approved by the Animal Care Ethic Committee of the Bulgarian Agency For Food Safety (BAFS) (protocol code 125 of 7 October 2020)

#### 2.2.1. Acute Toxicity in Mice

Acute toxicity was assessed in 36 female mice after oral (p.o.) and intraperitoneal (i.p.) administration of the compounds using a simplified method of Lorke [26] with slight modifications. Three animals were used per dose, at 5 fixed-dose intervals, with 3000 mg/kg being the highest dose for both compounds and 500 mg/kg being the lowest. Due to the low solubility of the test compounds in water, they were solubilized with Tween 80 (0.1%) before application. The LD_50_ was calculated using the following equation: ${\mathrm{LD}}_{50}=\sqrt{\left(D_{0}\times D_{100}\right)}$, where D_0_ is the highest non-lethal dose and D_100_ is the lowest lethal dose.

Surviving animals were observed every 3 h for the first 24 h and once a day for up to 14 days. During this period, the behavior of animals and the basic activities related to their breeding (walking, running, climbing, wrestling, and social behavior in a cage) were observed. Food intake and water intake were monitored daily by the veterinarian. Animals’ responses to “external stimuli” (manipulation response, straightening reflex, clapping response, response to noise or light fluctuations, toe or tail squeezing reflex) were assessed. On day 14, they were euthanized after anesthesia with ketamine/xylazine (80/10 mg/kg, i.p.) and an examination of the internal organs for possible macroscopic abnormalities (organ color, consistency, neoplasms, etc.) was done.

#### 2.2.2. Sub-Acute Toxicity

The sub-acute toxicity effects were assessed after repeated (14 days) oral administration to male mice. Based on the LD_50_ value after oral administration (higher than 2500 mg/kg for both compounds), two doses of 125 mg/kg and 250 mg/kg (≈1/20 and 1/10 of the LD_50_) were selected for multiple administrations. The experiments were performed with male Jcl: ICR mice at the same age of 6 weeks and weighing approximately 30–35 g, in which the substances were administered daily for 14 days orally with a gastric tube at approximately the same time of the day. The compounds were solubilized with Tween 80 (0.1%) in distilled water and administered orally in a volume of 0.1 mL per 10.0 g. Animals were observed daily for behavioral changes and signs of toxicity. All results were compared with the positive control isoniazid (INH). 

#### 2.2.3. Experimental Design 

For the sub-acute toxicity tests, the animals were divided into 6 experimental groups of 6 mice (*n* = 6) in each. Group 1—control mice; Group 2—mice treated orally with isoniazid (INH) 50 mg/kg [27]; Group 3—mice treated orally with **3a** at a dose of 125 mg/kg (1/20 LD_50_); Group 4—mice treated orally with **3a** at a dose of 250 mg/kg (1/10 LD_50_); Group 5—mice treated orally with **3b** at a dose of 125 mg/kg (1/20 LD_50_); Group 6—mice treated orally with **3b** at a dose of 250 mg/kg (1/10 LD_50_). 

The weights of the experimental animals were measured with a laboratory balance on days 1, 3, 5, 7, 9, 11, and 13. On day 14, the animals were anesthetized with ketamine/ xylazine and decapitated. Blood for serum biochemical investigations was collected in tubes containing a clot activator. After centrifugation at 3000× *g* for 10 min, the serum was separated. For complete blood count blood was also taken in vacutainers after decapitation and assessed. White blood cells number (WBC), Red blood cells count (RBC, Er), platelets (PLT) count, hemoglobin (Hb) concentration, and hematocrit (Ht) were measured using commercial kits for semi-automated hematological analyzer (BC-2800 Vet, Mindray, China) following the instructions of the manufacturer. Serum biochemical parameters were assessed as follows. Blood glucose level (Glu), urea (U), creatinine (Cr), Uric acid (UA), total protein (TP), albumin (Alb), aspartate aminotransferase (AST), alanine aminotransferase (ALT), total bilirubin (T-Bil), and direct bilirubin (D-Bil) were measured using commercial kits for automated biochemical analyzer (BS-120, Mindray, China) following the instructions of the manufacturer. Livers were taken to assess oxidative stress and antioxidant status in the study groups. Livers, kidneys and small intestine were taken for histological analysis.

### 2.3. Pathomorphological Evaluation of Tissue Specimens

Tissues from the liver, kidney and small intestine of the mice from all groups were collected post-mortem and fixed in 10% buffered formalin for 48 h. fixed tissues were processed according to the classical paraffin method [28]. The cutting of the paraffin blocks was performed using a paraffin rotary microtome Leica RM 2255 at a slice thickness of 5 µm. The sections were stained with hematoxylin and eosin (H&E). Histological changes were examined and imaged with a Leica DM2500 light microscope equipped with a Leica MC120HD digital camera and also with Euromex BioBlue (Belgium) digital camera

### 2.4. Redox-Modulating Capacity 

#### 2.4.1. Lipid Peroxidation Inhibition Assay

Malondialdehyde as a marker of lipid peroxidation was determined by the formation of endogenous lipid peroxidation products, reacting with 2-thiobarbituric acid (TBARS), and detected spectrophotometrically (λmax = 532 nm) adapted by Mileva et al. [29]. The method is based on the reaction of thiobarbituric acid (TBA) with the final products of lipids oxidation and formation of malondialdehyde (MDA). For in vitro studies (Fe—induced lipid peroxidation) the tested drugs in a concentration range from 5 mg/mL to 0.0156 mg/mL were incubated for 37 °C in brain homogenate (mg protein/mL) in the presence of 10 mM FeCl_3_ and 10 mM ascorbic acid. After that to the samples was added 0.6 mL a mixture of trichloroacetic acid (TCA)—TCA:HCl:TBA in 2:1:2 ratio and boiled in a water bath. After cooling and centrifugation at 3000 nrp the obtained color complex was read spectrophotometrically at A_532_ nm against the appropriate blank. The results were expressed in percentage inhibition of lipid peroxidation (received MDA per mg protein content from the control).

For the in vivo experiments (spontaneous lipid peroxidation) the postnuclear homogenates of the liver (mg protein/mL) in 0.15 M KCl—10 mM potassium phosphate buffer, pH 7.2, were heated for 15 min at 100 °C in the presence of 40% TCA + 5N HCl + 2% thiobarbituric acid (2:1:2 *v*/*v*) for color developing. After cooling and centrifugation, the absorbance was read at 532 nm against dd H_2_O as blank. The values were expressed in nmoles malondialdehyde (MDA) per mg protein, with a molar extinction coefficient of 1.56 × 10^−5^ M^−1^cm^−1^.

#### 2.4.2. Protein Content

The Biuret method based on a colorimetric test for total protein was used to determine the protein content. The Assay Kit was purchased from Cromatest/Lineal Chemicals REF No. 1153020.

#### 2.4.3. Measurement of the Amount of Total Glutathione

According to Rahman et al. [30] total glutathione (tGSH) concentration was determined. In the reaction between GSH and 5,5′-dithiobis-2-nitrobenzoic acid (DTNB), the rate of formation of colored substance, 5′-thio-2-nitrobenzoic acid (TNB), is proportional to the concentration of tGSH in the sample. The maximal absorption of colored TNB is at 412 nm. The concentration of tGSH was calculated using oxidized glutathione as a reference standard and was expressed as ng/mg protein.

#### 2.4.4. Enzyme Activity of Superoxide Dismutase (SOD)

SOD activity was measured by the method of Beauchamp and Fridovich [31]. Superoxide anion radicals generated photochemically for 7 min, reduced nitro blue tetrazolium (NBT) obtaining insoluble formazan in violet color. The reduction of NBT inhibition in the presence of different enzyme concentrations was read at 560 nm vs. control of reaction mixture. The amount of enzyme performing 50% inhibition of NBT reduction is accepted as a unit of activity (U/mg protein).

#### 2.4.5. Measurement of the Glutathione Peroxidase Activity

The Glutathione Peroxidase Cellular Activity Assay Kit (Cat. No. CGP1) and Glutathione Reductase Assay Kit (Cat. No. GRSA) were used to measure the activities of glutathione-related enzymes.

#### 2.4.6. DPPH Assay 

DPPH (2,2′-diphenyl-1-picrylhydrazyl radical) assay analysis of pure compounds was conducted by the method described by Brand-Williams et al. [32]. First, 500 μL of the test solutions were added to 500 μL of a freshly prepared solution of 0.1 mM DPPH in methanol and incubated in the dark for 30 min. Then the absorbance at 517 nm (A517) was measured against a mixture of DPPH solution and methanol (1:1) as a control. 

#### 2.4.7. ABTS•+ Assay

ABTS (2,2′-azino-bis (3-ethylbenzthiazoline-6-sulphonic acid) (7.0 mM) was mixed with potassium persulfate (2.45 mM) to obtain the cationic radical (ABTS•+) [33] The solution was diluted in methanol (2 mL ABTS•+ + 58 mL methanol). The absorption at 743 nm of the resulting working solution was 1.1 ± 0.02 AU. Then, 1.425 mL of ABTS•+ working solution was added to 75 μL of the test drugs, and the absorption at 743 nm was measured after 15 min of incubation at 37 °C against methanol. A blank containing 75 μL of water instead of the test extract also was measured against methanol. 

All data are presented as IC_50_, which gave 50% inhibition of the activities and compared against the activity of Trolox—a common antioxidant, at the same concentrations. 

### 2.5. Intestinal Permeability

The intestinal permeability of isoniazid and the newly synthesized derivatives was measured by PAMPA Permeability Analyzer (pION Inc., Billerica, MA, USA) following the Double-Sink™ Protocol [34]. PAMPA assay kits were purchased from pION Inc. According to the protocol, the donor compartment of the “sandwich” is loaded with compound solutions adjusted to three different pH values of 5.0, 6.2, and 7.4. The acceptor wells are filled with an ASB of fixed pH 7.4, containing chemical scavengers that simulate the effect of serum proteins. In the central circulation, pH 7.4 is constantly maintained, and the blood flow, as well as the serum proteins, support the one-directional process of absorption. Reference spectra (ketoprofen, antipyrine, and ranitidine) were used to assess the transfer of compounds from the donor to the acceptor phase. The permeation ability was estimated based on permeability coefficient values Pe (10^−6^ cm/s), presented in logarithmic terms (−logPe). Values of −logPe < 5 were accepted as indicative of highly permeable compounds. When −logPe acquired values between 5 and 6, compounds were considered as medium permeable, and if −logPe > 6–as low permeable [35]. Experiments were performed in triplicate and permeation coefficients Pe (−logPe values, respectively) are calculated using PAMPA Explorer Command Software (Version 3.8).

### 2.6. Statistical Analysis

The statistical program ‘MEDCALC’ was used for the analysis of the in vivo data. The results are expressed as mean ± SD for six animals in each group. The significance of the data was assessed by the nonparametric Mann–Whitney *U* test. Values of *p* 0.05 were considered statistically significant.

## 3. Results and Discussion

### 3.1. Chemistry

The series of 28 new aroylhydrazones which were synthesized previously [22,23] demonstrated significantly low minimal inhibitory concentrations. The synthetic strategy for these hydrazone derivatives has been reported previously and their spectral analyses were carried out by the assigned structures [22,23].

Two of them with low cytotoxicity and a highly selective index **3a** and **3b** were selected and shown in Figure 1.

### 3.2. Acute Toxicity in Mice

Dose intervals and symptoms observed after intraperitoneal administration of **3a** and **3b** are presented in Table 1 and Table 2.

Based on the results, LD_50_ for **3a** was calculated as follows:

${\mathrm{LD}}_{50}=\sqrt{D_{0}\times \;D_{100}}$ = $\sqrt{1000\times 1500}$ = 1224.7 mg/kg for i.p. administration.

No mortality was observed with oral administration at the highest dose of 3000 mg/kg, ie. LD_50_ > 2500 mg/kg. The resorption index (IR) was calculated as follows:

IR = LD_50_ i.p./LD_50_ p.os. × 100 = 1225/2500 × 100 ≈ 49%

The doses used for subacute (14 days) oral treatment were respectively parts of the LD_50_ for oral administration, i.e., 1/10 LD_50_ = 250 mg/kg and 1/20 LD_50_ = 125 mg/kg

Based on the results, LD_50_ for **3a** was calculated as follows:

${\mathrm{LD}}_{50}=\sqrt{D_{0}\times \;D_{100}}$ = $\sqrt{2000\times 3000}$ = 2449.49, i.e., LD_50_ > 2000 mg/kg for i.p. administration.

No mortality was observed with oral administration at the highest dose of 3000 mg/kg, i.e., LD_50_ > 2500 mg/kg. The resorption index (IR) is

IR = LD_50_ i.p./LD_50_ p.os ×100 ≈ 2450/2500 × 100 ≈ 98%

The doses used for subacute (14 days) oral administration were respectively: 1/10 LD_50_ = 250 mg/kg and 1/20 LD_50_ = 125 mg/kg.

The animals that survived the acute toxicity tests were observed once daily for up to 14 days. During this period, changes in social behavior in the cage and the reactions of animals to “external stimuli” were not observed. No changes in the skin, fur, eyes, mucous membranes, secretion, and autonomic activity (lacrimation, piloerection, pupil size changes, or unusual respiratory movements) were noticed. No changes in gait or response to the manipulation were observed, as well as the presence of clonic or tonic movements or strange behavior (e.g., aggression, walking backward). Neurotoxic effects such as sensory reactivity to various types of stimuli (auditory, visual and proprioceptive) have not been reported. There is no change in the amount of food and water intake. On day 14 after acute toxicity, all other living animals were euthanized. On day 14, the animals were anesthetized with ketamine/xylazine and decapitated. Macroscopic examination at autopsy does not show gross visible changes or lesions in the vital organs. No changes in the size, color, and consistency of the lungs, liver, heart, kidneys, stomach, spleen, and intestine were observed. No abnormalities in the morphology of the gonads and brain were detected.

Thus, acute toxicity studies of the compounds showed relatively low oral toxicity and slightly more parenteral toxicity. According to the Hodge and Sterner scale [36], the investigated compounds could be classified as slightly toxic when they were administered orally to female mice (LD_50_ > 2000 mg/kg b.w.). These results confirmed the data we obtained previously with compound **3a** [37]. Weight gain and the absence of statistically significant changes in the hematological, biochemical, and pathomorphological parameters in the blood, livers, small intestines, and kidneys of rats treated for 14 days with the test compounds showed good tolerability of the experimental animals to the investigated compounds **3a** and **3b** used at the appropriate doses.

The results of our toxicity tests are similar to the results obtained by Dragostin et al. [38,39] who found that the isonicotinoylhydrazones they synthesized had a significantly improved pharmacotoxicological profile compared to that of INH.

### 3.3. Sub-Acute Toxicity of ***3a*** and ***3b*** after 14 Days of Oral Administration of the Tested Compounds

**Figure 2:**
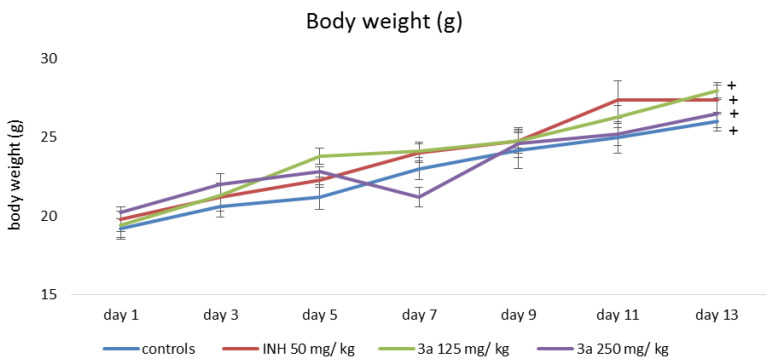
Changes in the body weight of animals treated with INH and **3a**; ^+^ *p* < 0.05 vs. 1st day of the experimental period.

**Figure 3:**
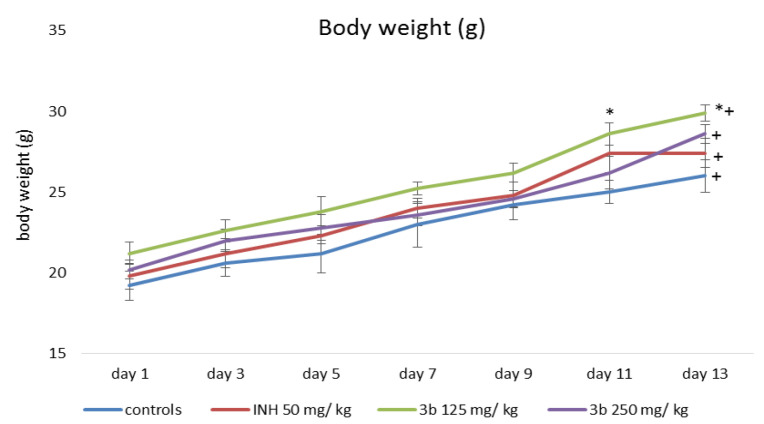
Changes in the body weight of animals treated with INH and **3b**; * *p* < 0.05 vs. control; ^+^ *p* < 0.05 vs. 1st day of the experimental period.

Changes in animal body weight during the experimental 14-day period are shown in Figure 2 and Figure 3. When measuring the body weight of animals from all experimental groups, statistically significant weight gain was found. At the end of the experimental period the animals treated with 125 mg/kg **3a** and **3b**, gained the most significantly with 41% and 44%, respectively, compared to the weight measured at the beginning of the experimental period.

#### Complete Blood Count (CBC) and Biochemistry in the Blood of Mice

Table 3 presents the results of the CBC analysis of the experimental animals. Administration of isoniazid resulted in a statistically significant reduction in the level of erythrocytes, hemoglobin, and hematocrit by 28%, 21%, and 20%, respectively, compared to the control group and the reference values for mice. Leukocytes were increased by 70% compared to controls but remained within the reference range for mice. No platelet abnormalities were observed after 14 days of oral isoniazid. In animals treated with both doses of **3a**, no statistically significant deviations from the reference values were observed, indicating no adverse effect of the test substances on haematopoiesis.

Analysis of the biochemical parameters (Table 4) demonstrated a statistically significant increase in the parameters related to liver and kidney function after 14 days of oral administration of isoniazid. ASAT and ALAT transaminases increased by 52% and 67%, respectively, compared to the values of the control animals, as well as compared to the reference limits for mice. Total and direct bilirubin levels were also 40% and 48% higher than controls after 14 days of isoniazid administration, respectively. Albumin and total protein statistically significantly decreased their levels, by 23% and 26%, respectively, compared to the controls and reference values, indicating impaired synthetic liver function. Urea and creatinine increased by 49% and 29%, respectively, compared to controls, which is associated with impaired renal function. No blood sugar deviations were observed in any of the experimental groups of animals compared to the reference values. The tested substances did not lead to statistically significant deviations in the studied biochemical parameters, compared to the reference values and the control group.

### 3.4. Pathomorphological Evaluation of Tissue Specimens

#### 3.4.1. Liver

**Figure 4:**
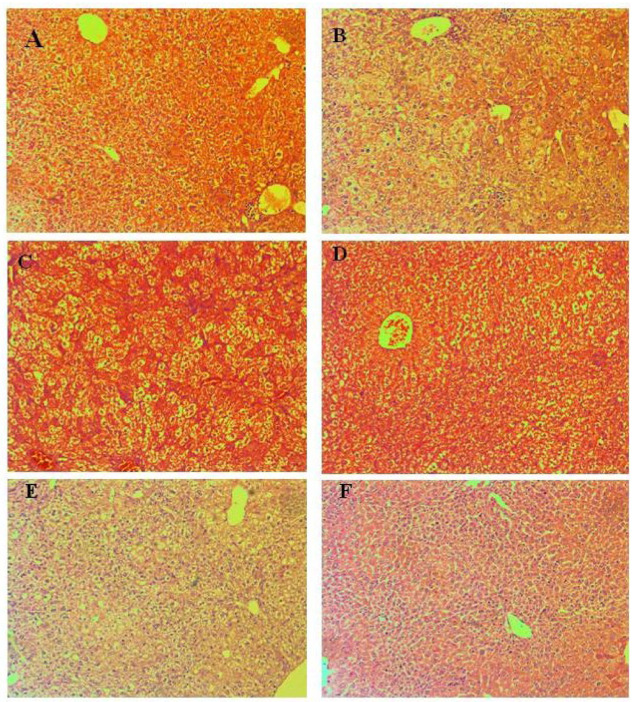
Pathomorphological findings in the liver in mice after oral administration of Isoniazid (INH) and its derivatives. Legend: (**A**). Control group—not treated, (**B**)-INH, 50 mg/kg; (**C**)—**3a**, 125 mg/kg, (**D**)—**3a**, 250 mg/kg; (**E**)—**3b**, 125 mg/kg; (**F**)—**3b**, 250 mg/kg, Magnification of the field 100×.

Liver findings show the manifestation of individual changes (Figure 4) and the absence of a formed pathological organ profile. Lobular structure, lack of remodeling zones, intrahepatic cholestasis in individual cells, and regenerative activity within tissue-specific parameters were found in the organ parenchyma. The parameters of the biliary system include correctly presented structures and the absence of portal canalicular proliferative changes. Circulatory changes in the organs include passive venous hyperemia, dilatation of the sinusoidal spaces, and preserved hepatic lamellae. Large and medium-sized organ veins show a preserved histological structure, without signs of intimal and mural changes. Isolated intrahepatic cholestasis was detected. Lobular inflammatory process and hepatocyte loss are not reported in groups treated with Isoniazid 50 mg/kg, compounds **3a** 125 mg/kg, and **3a** 250 mg/kg. Manifestations of regenerative response are isolated in single lobules. The lack of lobular inflammatory process is accompanied by foci of portal and periportally presented minimal inflammatory process. The outbreaks affect a small number of portal spaces and show a limited area. Extension of the inflammatory process to the portal and intermediate zones (zones 2 and 3 of the liver lobes)—a positive finding for limited hepatitis is reported in groups treated with **3b** 125 mg/kg, **3b** 250 mg/kg. The histological profile shows minimal degenerative changes in hepatocytes with the zonal distribution. In the group E (**3b** in dose 125 mg/kg) signs of increased ballooning degeneration of hepatocytes varying in the range of 8–18% were found, compared with the detected lower interval from 2–8% in the group F (**3b** in dose 250 mg/kg). In the other experimental groups, signs of minimal hepatocyte ballooning degeneration were found. Minimal steatosis (3–5%) was reported, as the representation is from small vesicular forms. The histological profile shows isolated degenerative changes in hepatocytes with a non-zonal distribution. The changes are not associated with toxic organ manifestations. Some cells show cytoplasmic changes and centrally located nuclei in intracytoplasmic septation. The finding is not repeated and is not accompanied by loss of membrane organelles. The possibility of metabolic overload of some types of membrane organelles is not accompanied by increased turnover of these organelles and vacuoles of autophagy are observed as a single finding. No signs related to initiated fibroblastic process (initiated organ collagenization and fibrosis) were found in groups treated with Isoniazid 50 mg/kg, **3a** 125 mg/kg, **3a** 125 mg/kg, **3b** 125 mg/kg, **3b** 250 mg/kg.

Lesions of increased ballooning degeneration were seen in the group with **3b** 3000 mg/kg oral and intraperitoneal administration varying in the range of 2–12% and in the range of 2–14% respectively. Scars related to initiated fibroblastic process (initiated organ collagenization) are established as an isolated finding of minimal perisinusoidal and portal collagenization in groups with **3b** 3000 mg/kg in oral and intraperitoneal administration.

#### 3.4.2. Kidneys

**Figure 5:**
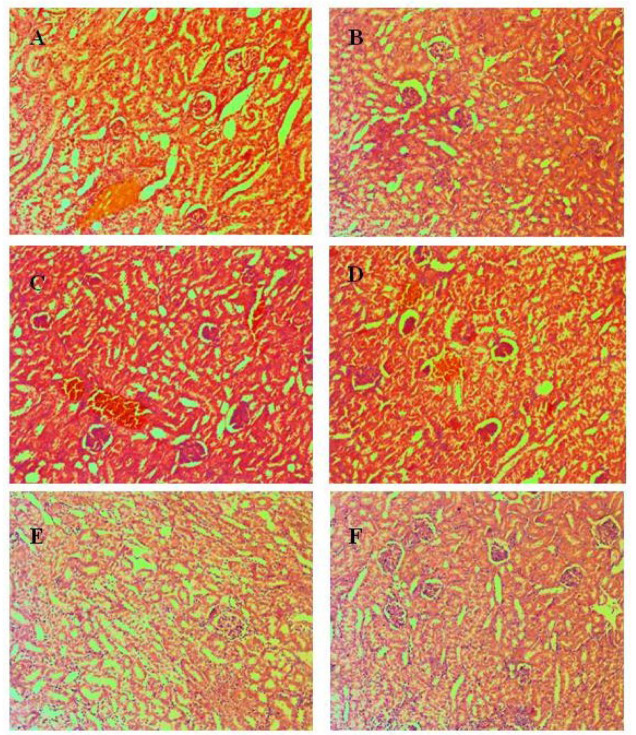
Pathomorphological findings in the kidney in mice after oral administration of Isoniazid (INH) and its derivatives. Legend: (**A**). Control group—not treated, (**B**)—INH, 50 mg/kg; (**C**)—**3a**, 125 mg/kg; (**D**)—**3b**, 250 mg/kg; (**E**)—**3b**, 125 mg/kg; (**F**)—**3b**, 250 mg/kg; Magnification of the field 100×.

The changes in the kidneys do not show an organ pole topography. Microscopical findings in the kidneys of treated mice are presented in Figure 5. The reported histological findings contain features compatible with normal histological architecture, without being associated with concomitant pathological conditions. The vascular representation is histologically consistent and is not accompanied by vascular fibrointimal changes and luminal reduction. No findings of tubulitis, tubular atrophy, and glomerulitis. The cortical labyrinth is correctly represented by varying luminal areas in the proximal tubules at the height of the upholstered cells in the reference norm. The curved parts of the distal tubules have a lower upholstered epithelium, wide luminal segments, and preserved histological structure. Elements of glomerular and extraglomerular mesangium are visualized in separate areas and show no signs of proliferation. Straight segments of the proximal tubules are found in single areas. The outer zone of the medullary part shows a narrow variation in thickness in organs from the exposed groups. The visualization of the inner zone shows collecting ducts with preserved histological structures. Minimal variations in the presented periglomerular interstitium. The excretory structures of the organs (sinus of the kidneys and the ampullary part of the renal pelvis) have a preserved histological structure at the exposure doses. In Control group A, the cortical labyrinth has varying luminal areas in the proximal tubules at the height of the upholstered cells in the reference norm. In group B (INH in dose 50 mg/kg), the curved parts of the distal tubules have a lower upholstered epithelium, wide luminal segments, and preserved histological structure. In group C (**3a** in dose 125 mg/kg), minimal circulatory lesions presented with edema in the interstitium are observed. In group D (**3a** in dose 250 mg/kg), no findings of tubulitis, tubular atrophy, and glomerulitis. In group E (**3b** in dose 125 mg/kg), the cortical labyrinth is correctly represented by varying luminal zones. In group F (**3b** in dose 250 mg/kg), minimal variations in the presented periglomerular interstitium are observed.

Straight segments of the proximal tubules are found in single areas in dose 3000 mg/kg **3b**, oral administration. The outer zone of the medullary part in the kidney shows a narrow variation in thickness for **3b** in dose 3000 mg/kg, intraperitoneal administration.

#### 3.4.3. Small Intestine

**Figure 6:**
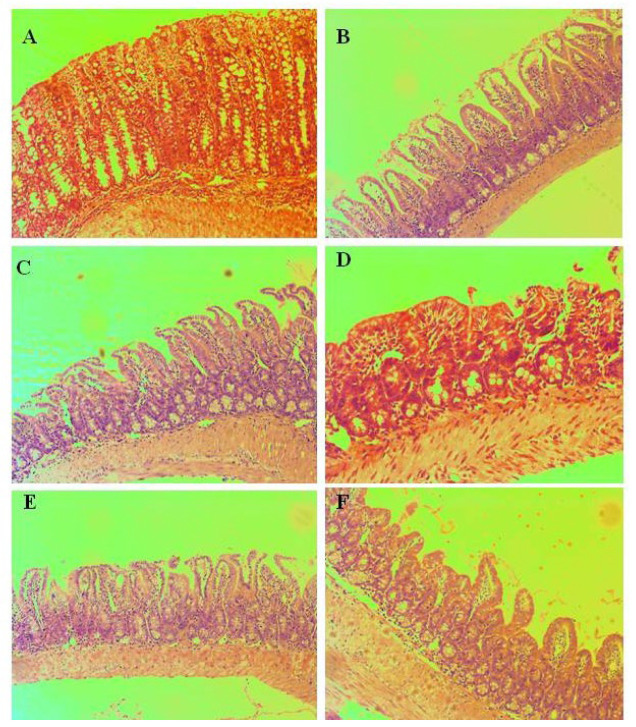
Pathomorphological findings in the small intestine in mice after oral administration of Isoniazid (INH) and its derivatives. Legend: (**A**). Control group—not treated, (**B**)—INH, 50 mg/kg; (**C**)—**3a**, 125 mg/kg; (**D**)—**3a**, 250 mg/kg; (**E**)—**3b**, 125 mg/kg; (**F**)—**3b**, 250 mg/kg; Magnification of the field 100×.

Pathomorphological findings in small intestines of mice after oral administration of Isoniazid and its derivatives are shown in Figure 6. In Control group A, the leukocytes did not show a high density and pronounced representation. In group B (INH, 50 mg/kg), there were no defects in the epithelium, reaching and exceeding the muscular layer of the epithelium (lamina muscularis mucosae). Changes in the crypt architecture were isolated. The villous-crypt ratio was most often 2:1. This determines a score of 1 and a low (minimum) degree of villous dullness. In group C (**3a**, 125 mg/kg), there was a low degree of severity of the inflammatory process. In group D (**3a**, 250 mg/kg), non-parallel crypts were found in each of the treated groups. Variable diameters of crypts were found, such as a very low frequency find. In group E (**3b**, 125 mg/kg), there are no findings of viliform, hypertrophic areas and protrusions to the lumen, which lead to a luminal reduction. In group F (**3b**, 250 mg/kg), no signs of aggression of inflammatory cells between crypt epithelial cells were observed. Neutrophil leukocytes in crypt lumens were single.

#### 3.4.4. Evaluation of Inflammatory Changes

The density of leukocytes estimated on the area of the own plate (assessment on the fields of a high-power magnification) did not show high density and pronounced representation. The assessment of the density in the treated groups compared to the control is minimal (below 10% and grade 1). In individual animals, the density reached 10%, but without dispersion of inflammatory cells, and grade 2 was not supported by a sufficient amount of histological evidence. In the treated groups there was a low degree of severity of the inflammatory process. Expansion of inflammatory infiltration included mucosal involvement (grade 1) and mucosal and submucosal involvement (grade 2). The affected areas have a low increase compared to the control animals. The increase in the exposure dose showed similar results and low variability. The results obtained do not differ from those in inflammatory processes with low intensity and affecting limited structures of the intestinal wall.

#### 3.4.5. Evaluation of Epithelial Changes

In the treated groups, the increase in epithelial cells in the longitudinal crypts compared to the initial number of epithelial cells in the crypt showed variations from 12% to 19%. No value above 25% was reported in any of the groups and the grade for hyperplastic changes was 1. The loss of Goblet cells is estimated as a reduced number of Goblet cells compared to the initial number of Goblet cells in the crypt. In the treated groups the variation in this indicator is from 9% to 14%. The interval in the control group was 8–10%. The limit of 20% was not exceeded, leading to a minimum degree of Goblet cell loss and a score of 1. No signs of aggression of inflammatory cells between crypt epithelial cells were reported, and neutrophil leukocytes in crypt lumens were single. These changes were not associated with the loss of superficial epithelium. The findings lead to a score of 0 on the indicators cryptitis, crypt-abscesses, and erosions (erosion changes).

#### 3.4.6. Assessment of Mucosal Architecture

In the treated groups, no defects in the epithelium were reached, reaching and exceeding the muscular layer of the epithelium (lamina muscularis mucosae). Changes in crypt architecture were isolated. Non-parallel crypts were found in each of the treated groups. Variable diameters of crypts were found, such as a very low frequency find. These findings did not show a correlation. No recurrent bifurcations and branched crypts were found in any of the groups. The changes are not accompanied by the loss of crypts. The scores on these indicators are low and do not form a representative sum-forming score. Isolated pseudopolyps were found in the treated groups. No findings of viliform, hypertrophic areas and protrusions to the lumen, which lead to a luminal reduction, were found in the studied areas.

#### 3.4.7. Assessment of Villous Dullness

In the treated groups the villous-crypt ratio was most often 2:1. This determines a score of 1 and a low (minimum) degree of villous dullness. No finding of villous atrophy was found in the exposure groups. The obtained cumulative estimates did not show parameters for changes in the mucosal architecture in the indicated exposure doses.

### 3.5. Redox-Modulating Capacity

For a complete pharmacological profile of newly synthesized compounds and to elucidate the mechanisms of their toxicity, we studied the redox-modulating capacity of the investigated substances: (i) radical-scavenging and metal-chelating in model chemical systems in vitro; (ii) influencing Reactive oxygen species (ROS)-mediated homeostasis in the liver of experimental animals.

The DPPH (2,2-diphenyl-1-picryl-hydrazyl-hydrate) free radical assay usually involves a hydrogen atom transfer reaction [37]. DPPH radical scavenging test is a sensitive antioxidant assay and depends on substrate polarity. Our results showed that **3a** and **3b** can quench DPPH free radicals and convert them to a colorless product, resulting in a decrease in absorbance at 517 nm. The scavenging activities were expressed as IC_50_ (mg/mL). Based on the results in Table 5, there is a significant decrease (*p* < 0.01) in the concentration of DPPH radicals due to the scavenging ability of **3a** and **3b** versus blank system, but the effects are significantly weaker than that of Trolox (6-hydroxy-2,5,7,8-tetramethylchroman-2-carboxylic acid, water-soluble analog of vitamin E). 

The better activity of substance **3a** towards both radicals is probably due to the polar bonded hydrogen in its molecule (Figure 1). However, both radicals do not found naturally, so there exists a possibility that the results in vitro are not directly relevant to any biological function in vivo.

**Figure 7:**
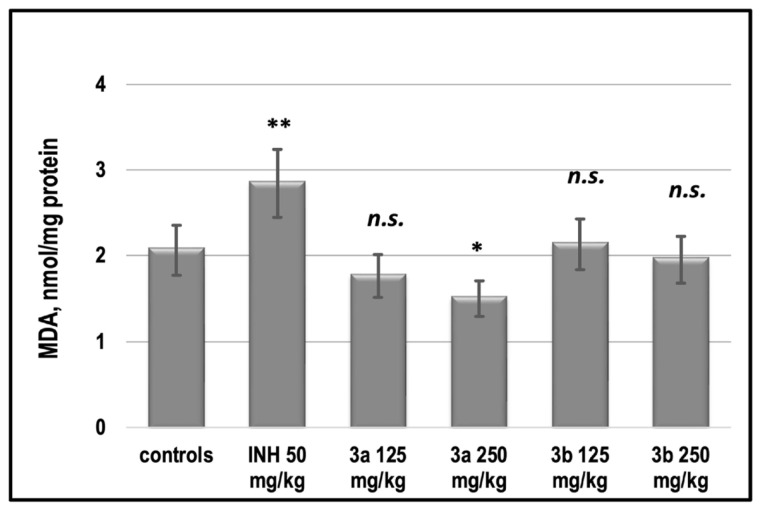
Endogenous content of MDA in the liver homogenate of experimental groups. * *p* < 0.05 vs. controls; ** *p* < 0.01 vs. controls; n.s.—nonsignificant vs. INH. Results are expressed as mean ± SD (*n* = 6). The significance of the data was assessed using the nonparametric Mann–Whitney *U* test. Values of *p* ≤ 0.05 were considered statistically significant.

The effect of INH on the level of lipid peroxidation, determined by the amount of TBARs in the liver homogenate is shown in Figure 7. After the application of INH, the level of MDA increased by more than 35%. Supplementation of mice with INH and **3a** and **3b** according to the therapeutic scheme in both doses led to the restoration of MDA to the control levels. Our results showed that in in vivo conditions substance **3a** showed better protection against iron-induced lipid peroxidation (Figure 7) compared to the in vitro model system (Table 5).

**Figure 8:**
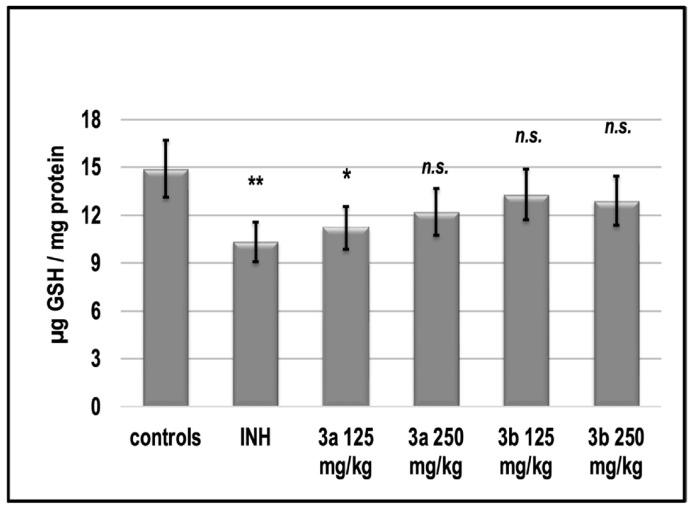
Endogenous content of GSH in the liver homogenate of experimental groups. * *p* < 0.05 vs. controls; ** *p* < 0.01 vs. controls; n.s.—nonsignificant vs. controls. Results are expressed as mean ± SD (*n* = 6). The significance of the data was assessed using the nonparametric Mann–Whitney *U* test. Values of *p* ≤ 0.05 were considered statistically significant.

The effect of INH on the level of endogenous content of glutathione (GSH) in the liver homogenate of experimental groups is shown in Figure 8.

**Figure 9:**
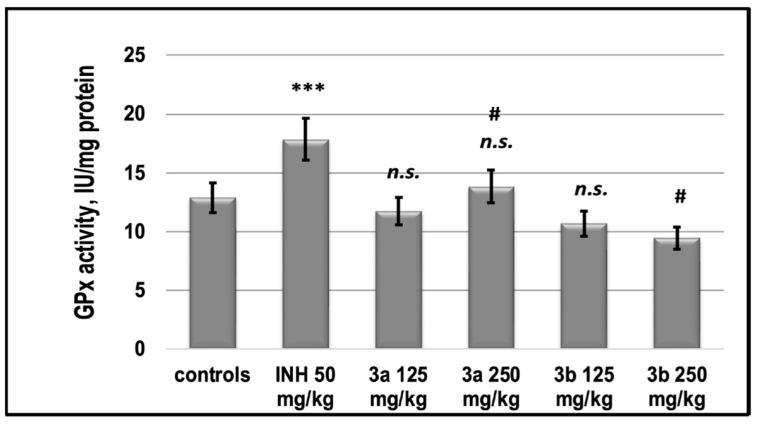
The activity of Glutathione peroxidase in the liver supernatant of experimental groups. *** *p* < 0.001 vs. controls; # *p* < 0.01 vs. INH 50 mg/kg; n.s.—nonsignificant vs. controls. Results are expressed as mean ± SD (*n* = 6). The significance of the data was assessed using the nonparametric Mann–Whitney *U* test. Values of *p* ≤ 0.05 were considered statistically significant.

In parallel with the decrease in MDA, we found that INH decreased the level of total GSH. The compound **3a**, as well as **3b**, in both doses, did not significantly increase the level of GSH. The activity of SOD was increased significantly by INH with 25%. Both compounds **3a** and **3b** decreased the activity of the enzyme even under the control values (Figure 9).

INH increased the activity of GPx compared to controls by 35%, **3a** in both supplemented doses, as well as **3b** restored the control values of GPx (Figure 9).

Reactive oxygen species (ROS), such as superoxide-anion radical (O2-), hydroxyl radical (HO·), hydroperoxyl radical (HOO-), hydrogen peroxide (H2O2), etc. are normally produced in cells during metabolic processes. ROS are generated in the cellular mitochondria, in the mitochondrial electron transport chain from intracellular sources, for example, nicotinamide adenine dinucleotide phosphate (NADPH) oxidase, enzymatic processes such as activation of cytochrome P-450, xanthine oxidase, arachidonic acid metabolism, etc. [40,41,42].

Overproduction of ROS initiated by extracellular sources, including xenobiotics such as chemotherapeutic drugs, can pose a problem. The production of ROS above acceptable levels can cause damage to cellular macromolecules such as DNA, membrane lipids, and proteins, and disrupt their function. The equilibrium level of physiologically generated ROS is controlled by enzymatic and non-enzymatic intracellular antioxidant systems, which are associated with different functional pathways [43].

This gives rise to a need to investigate the antioxidant/prooxidant properties of newly synthesized compounds and their ability to influence the redox state of cells and/or possibly prevent prooxidant-mediated oxidative damage.

MDA is an endogenous genotoxic product, considered a biochemical marker of enzymatic and/or ROS-mediated lipid peroxidation. MDA is the most popular indicator of oxidative damage to cells and tissues. It can lead to cross-linking polymerization of biological macromolecules and thus realize its damaging role as a serious cytotoxic and genotoxic factor. MDA content is usually used as a basis for evaluating the degree of lipid peroxidation and reflecting the level of damage to cells and tissues from the effects of pro-oxidant agents [44]. The compound **3b** at both doses did not statistically significantly change MDA content in the liver, which could be due to its low pro-oxidant activity.

**Figure 10:**
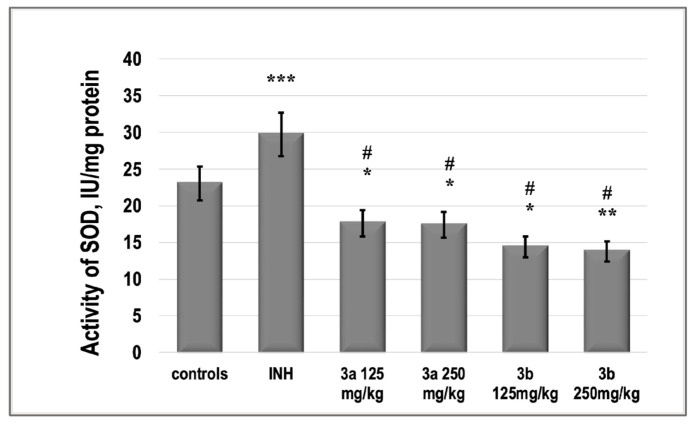
The activity of SOD in the liver supernatant of experimental groups. *** *p* < 0.001 vs. controls; ** *p* < 0.01 vs. controls; * *p* < 0.05 vs. controls; # *p* < 0.001 vs. INH 50 mg/kg. Results are expressed as mean ± SD (*n* = 6). The significance of the data was assessed using the nonparametric Mann–Whitney *U* test. Values of *p* ≤ 0.05 were considered statistically significant.

There is a set of free radical scavenging enzyme defense systems in the body, such as SOD, GSH-Px, and CAT, which synergistically scavenge superoxide radicals, hydroxyl radicals, and hydrogen peroxide, respectively, and maintain equilibrium levels of glutathione [45]. In this study, we found that both **3a** and **3b** did not statistically significantly alter the activities of the pro-oxidant enzymes GPx and SOD relative to those of untreated control animals, in contrast to INH, which increased them (Figure 9 and Figure 10). At the same time, the two newly synthesized analogs did not change the glutathione content in these groups compared to the control levels, while INH induced glutathione deficiency by about 30% (Figure 9).

The current in vitro study found that **3a** and **3b** can suppress Fe^2+^-asc.—induced lipid peroxidation in a model system can trap DPPH and ABTS radicals but is significantly weaker than Trolox (Table 5). Our results in vivo experiment showed that the tested compounds **3a** and **3b** can restore the oxidative stress markers. All of the applied doses of both substances had similar redox-modulating potential. We suppose, they can act as symptomatic drugs which can control the oxidative damages accompanying liver toxicosis in conditions of tuberculosis infection.

### 3.6. Gastrointestinal (GIT) Permeability

PAMPA results show that INH is impermeable at the given experimental conditions (−logPe > 6). The newly synthesized derivatives possess better permeability than NIH (Table 6), which is reflected in the values of −logPe. These are from one to two orders of magnitude lower for the test compounds. Compound **3b** possesses a high GIT permeability at all the tested pH values (5.0, 6.2, 7.4), (Table 6). The data also indicate that a change in pH does not induce noticeable variations in the permeability of compounds. This suggests pH-independent permeation through the GIT membranes and is theoretically supported by the physicochemical properties of compounds. They are all very weak acids existing in the unionized form at physiological conditions and have higher lipophilicity than INH (comparison relay on calculated logP values by SwissADME [23,46].

PAMPA is a valuable in vitro tool in the early stages of drug discovery and optimization [47]. It models the passive diffusion of compounds through an artificial membrane, mimicking the gastrointestinal barrier. The method is well suited for the prediction of gastrointestinal absorption because the majority of drugs diffuse passively through membranes [48,49,50]. In the present study, PAMPA was used as a simple and cost-effective approach for estimation the permeability potential of the new aroylhydrazone derivatives and presents a useful tool for an early stage ADME screening of orally administered compounds.

## 4. Conclusions

To conclude, the new aroylhydrazone compounds **3a** and **3b** were characterized for their: (i) acute and subacute toxicity in mice, (ii) redox-modulating capacity by in vivo and in vitro investigations, (iii) pathomorphological observation in the liver, kidney, and small intestine tissue specimens, and (iv) intestinal permeability. The compounds were classified as slightly toxic for oral and intraperitoneal administration: a histological examination of the different tissue specimens did not reveal toxic changes. At the same time, liver findings showed only isolated changes without a pathological organ profile. The in vitro antioxidant assays confirmed the ex vivo results. Structural modifications successfully improve the permeation profiles of INH derivatives. High GIT permeability at all tested pH values were demonstrated for both compounds and they possess better permeability than NIH. Weight gain and the absence of statistically significant changes in the hematological, biochemical, and pathomorphological parameters in the blood, livers, small intestines, and kidneys of mice treated for 14 days with both compounds showed good tolerability of the experimental animals to both investigated compounds used at the appropriate doses.

Thus, both compounds **3a** and **3b** are highly permeable with low toxicity and can be considered for further evaluation and/or lead optimization.

## Figures and Tables

**Table 1:** Acute intraperitoneal toxicity of **3a**.

Dose mg/kg b.w.	Lethality	Time of Occurrence of Fatal Outcome	Symptoms before Death Occur
3000	2/3 (67%)	After 20 h	Respiratory failure with long pauses, ataxia, piloerection, seizures, lethal outcome
2000	1/3 (33%)	After 24 h	Impaired coordination, rapid breathing, lethal outcome
1500	1/3 (33%)	After 30 h	Delayed reflexes, somnolence, lethal outcome
1000	0/3	-	-
500	0/3	-	-

**Table 2:** Acute intraperitoneal toxicity of **3b**.

Dose mg/kg b.w.	Lethality	Time of Occurrence of Fatal Outcome	Symptoms before Death Occurs
3000	1/3 (33%)	After 3 h	Impaired coordination, rapid breathing, clonic seizures, death
2000	0/3	-	-
1500	0/3	-	-
1000	0/3	-	-
500	0/3	-	-

**Table 3:** Complete blood count (CBC) after 14 days of administration of isoniazid (INH) 50 mg/kg, **3a** and **3b** at doses of 125 and 500 mg/kg.

Haemato Logical Parameters	Controls	INH 50 mg/kg	3a 125 mg/kg	3a 250 mg/kg	3b 125 mg/kg	3b 250 mg/kg	Reference Values
WBC × 10^9^/L	5.8 ± 0.34	9.9 ± 0.54 *	6.3 ± 0.36 ^+^	7.4 ± 0.44 *^+^	8.2 ± 0.28 *^+^	7.7 ± 0.33 *^+^	2.9–15.3
RBC × 10^12^/L	7.39 ± 0.6	5.35 ± 0.2 *	6.84 ± 0.3 ^+^	6.97± 0.3 ^+^	7.21 ± 0.16 ^+^	7.62 ± 0.24 ^+^	5.6–7.89
Hgb g/L	142 ± 7.6	112 ± 2.2 *	127 ± 5.4 ^+^	129 ± 3.8 ^+^	132.2 ± 4.2 ^+^	130.2 ± 3.7 ^+^	120–150
HCT%	41.3 ± 1.8	33.3 ± 2.1 *	37.2 ± 2.6 ^+^	38.2 ± 1.8 ^+^	39.6 ± 0.8 ^+^	40.1 ± 1.2 ^+^	36–46
PLT 10^9^/L	886 ± 126	789 ± 183	979 ± 238	638 ± 181	729 ± 203	864 ± 212	100–1610

* p < 0.05 vs. controls; ^+^ *p* < 0.05 vs. INH Results are expressed as mean ± SD (*n* = 6). The significance of the data was assessed using the nonparametric Mann–Whitney *U* test. Values of *p* ≤ 0.05 were considered statistically significant. Abbreviations: WBC, White blood cells; RBC, Red blood cells; PLT, platelets; Hb, hemoglobin; and Ht, hematocrit.

**Table 4:** Biochemical parameters in serum from experimental animals after 14 days of administration of INH and its newly synthesized derivatives **3a** and **3b** at doses 125 and 250 mg/kg.

Biochemical Parameters	Controls	INH 50 mg/kg	3a 125 mg/kg	3a 250 mg/kg	3b 125 mg/kg	3b 250 mg/kg	Reference Values
GLU mmol/L	7.2 ± 0.32	6.4 ± 0.47	6.3 ± 0.36	7.1 ± 0.24	6.8 ± 0.22	7.0 ± 0.16	4.2–7.5
UREA mmol/L	9.1 ± 0.12	13.6 ± 0.36 *	8.8 ± 0.28 ^+^	11.5 ± 0.22 ^+^	8.7 ± 0.26 ^+^	8.4 ± 0.23 ^+^	3.27–12.1
CREAT µmol/L	98.3 ± 2.3	126.6 ± 8.2 *	95.6 ± 6.6 ^+^	86.4 ± 5.6 ^+^	88.2 ± 3.1 ^+^	92.2 ± 4.4 ^+^	35–120
UA µmol/L	195 ± 18.9	196 ± 20.7	163 ± 23.4	194 ± 17.7	199 ± 14.6	201.3 ± 12	0–300
TP g/L	58.1 ± 2.2	43.2 ± 3.1 *	54.3 ± 2.6 ^+^	58.6 ± 3.6 ^+^	56.6 ± 4.2 ^+^	52.2 ± 5.1 ^+^	53–63
ALB g/L	27.9 ± 1.8	21.6 ± 1.7 *	26.6 ± 2.2 ^+^	27.3 ± 3.1 ^+^	28.5 ± 3.3 ^+^	28.2 ± 2.8	26–29
ASAT U/L	83 ± 4.5	126 ± 5.2 *	102 ± 3.6 ^+^	113 ± 4.1	112.1 ± 4.4 *	110 ± 3.1 *	65–122
ALAT U/L	58 ± 2.2	96.6 ± 3.1 *	62.2 ± 3.3 ^+^	71.4 ± 3.4 *^+^	77.2 ± 2.8 *^+^	71.2 ± 1.9 *^+^	55–80
T-Bil µmol/L	8.6 ± 0.42	12.0 ± 0.38 *	7.6 ± 0.44 ^+^	8.3 ± 0.28 ^+^	7.8 ± 0.23 ^+^	8.1 ± 0.32 ^+^	3.9–9.6
D-Bil µmol/L	4.4 ± 0.32	6.5 ± 0.44 *	3.4 ± 0.46 ^+^	4.9 ± 0.34 ^+^	5.1 ± 0.23 ^+^	3.8 ± 0.42 ^+^	0–6.8

* *p* < 0.05 vs. controls; ^+^ *p* < 0.05 vs. INH; Results are expressed as mean ± SD (*n* = 6). The significance of the data was assessed using the nonparametric Mann–Whitney *U* test. Values of *p* ≤ 0.05 were considered statistically significant. Abbreviations: Glu, glucose level; CREAT, creatinine; UA, Uric acid; TP, total protein; Alb, albumin; AST, aspartate aminotransferase; ALT, alanine aminotransferase; T-Bil, total bilirubin; and D-Bil, direct bilirubin.

**Table 5:** In vitro redox-modulating properties of **3a** and **3b** compared to Trolox. Lipid peroxidation was tested in a model system of brain homogenate, as described in Materials and Methods.

	Parameter	Fe^2+^-asc.—Induced LP in Liver, IC_50_ [mg/mL]	DPPH Scavenging, IC_50_ [mg/mL]	ABTS Scavenging, IC_50_ [mg/mL]
Substance				
**3a**		76.29 *	47.5 *	89.47 *
**3b**		85.14 *	18.69 *	40.35 *
Trolox		18.2	8.92	24.8

DPPH—(2,2′-diphenyl-1-picrylhydrazyl radical) assay, ABTS—(2,2′-azino-bis (3-ethylbenzthiazoline-6-sulphonic acid) assay. * *p* < 0.01 vs. blank system.

**Table 6:** GIT permeability of INH and the newly synthesized derivatives.

Compound Code	−logPe, GIT PAMPA		
	pH = 5.0	pH = 6.2	pH = 7.4
INH	6.226	6.591	6.594
**3a**	4.354	4.366	4.299
**3b**	4.158	4.137	4.150

GIT—gastrointestinal tract, INH—isoniazid.

## Data Availability

All obtained data are presented in this article.
